# The Hospitals and Vivisection

**Published:** 1897-05-15

**Authors:** 


### THE INSTITUTIONAL WORKSHOP.

### THE HOSPITALS AND VIVISECTION.

THE following is quoted from *The Zoophilist* for May 1st:—

HOSPITALS, MEDICAL SCHOOLS, AND VIVISECTION.

A circular letter having been addressed, by direction of the committee of the Victoria Street Society, to the members of the Council of the Prince of Wales's "Diamond Jubilee Hospital Fund," Mr. Henry C. Burdett, one of the members of that Council, replied by a letter to the Secretary on the subject. In this letter he stated that he had "been actively interested in hospitals of every class for nearly thirty years, and had resided in some of the principal hospitals of this country, metropolitan and provincial, with medical schools attached and without, during fifteen years of that period," yet he had never known a case of experimentation on patients, or of grants from hospital funds to a medical school where vivisection was practised. He therefore asked for "the names of all the hospitals (1) where the patients or any of them are treated as subjects for experimentation apart from the treatment of their maladies; and (2) the names of the medical schools attached to hospitals, which receive grants from the hospital funds, in which the practice of vivisection is carried on." He also pronounced it "ridiculous nonsense" for Mr. Coleridge to write to the *Times* and "describe laboratories attached to hospitals where 'hapless dumb animals daily and hourly are slowly cut up alive without even the pretence of anæsthetics,'" and asked to be supplied with the names of the laboratories referred to.

The Secretary being temporarily disabled, the Hon. Stephen Coleridge, who was also directly attacked, on the 25th of March, wrote to Mr. Burdett as follows:—

HON. STEPHEN COLERIDGE TO MR. H. C. BURDETT.

SIR,—I have seen a letter of yours of the 15th of March in which you ask two questions of Mr. Bryan, of Victoria Street. With the first I am not concerned, with the second I am. You want to know the names of the medical schools that have received grants from hospital funds? In the report of the London Hospital for 1896-7 you will find it stated that the school practically owes its existence to the hospital funds, as the cost of erecting the original building, "which amounted to more than £10,000, was borne by the Governors of the hospital," and on page 8, following the history of the school down to a later date, it appears that £15,000 more was "lent" by the hospital to remodel the school buildings. In an advertisement of this hospital in the *Lancet* of May 13th, 1876, there appear extracts from the minutes of the meeting of the house committee, which are quoted as follows: "It was unanimously resolved that with a view to the improvement of the London Hospital Medical School . . . . it be recommended to the Governors that during the next three years the present grants to the college, amounting to £290, be replaced by a grant of £2,000 per annum." Vivisection has certainly been carried on in that school erected, supported, and remodelled with money granted and lent out of hospital funds. I can also refer you in the case of Guy's Hospital to a letter issued by the Governors, in which they say: "The Governors have given very substantial proof of the interest they take in the school by the expenditure of many thousands of pounds within the last few years." This was in October, 1880, at which time three persons held licences to vivisect in that school, and one of them held an additional certificate dispensing with the obligation to kill the animal before recovering from anæsthetics. I dare say if you pursue your researches in this direction you will find evidence of a similar nature with respect to other hospitals without my assistance. In writing my letter to the Prince of Wales it did not seem to me unlikely that what had certainly happened in the past might happen in the future, and I asked for some pledge that this Jubilee Fund, at any rate, might not be diverted from the relief of human suffering to the promotion of animal torture. To many people this does not seem a very unreasonable request. I am sorry you should think it necessary to endorse the hysterical personalities of the *British Medical Journal*.— I remain, yours faithfully, STEPHEN COLERIDGE.

7, Egerton Mansions, S.W.

MR. H. C. BURDETT TO HON. STEPHEN COLERIDGE.

The Lodge, Porchester Square, W.
27th March, 1897.

SIR,—I have to acknowledge receipt of your letter of the 25th instant. I will cause the statements in regard to the London and Guy's Hospitals contained therein to be thoroughly inquired into at once. I have, however, to point out that you have ignored the question I asked in my letter of the 15th instant which affects you. That question contains a request to be supplied with the names of the laboratories which you stated in the *Times* to be attached to hospitals where "hapless dumb animals daily and hourly are slowly cut up alive without even the pretence of anæsthetics."— I am, Sir, yours faithfully,

HENRY C. BURDETT.

The Hon. Stephen Coleridge,
7, Egerton Mansions, South Kensington.

HON. STEPHEN COLERIDGE TO MR. H. C. BURDETT.

7, Egerton Mansions, South Kensington.
29th of March, 1897.

SIR,—I have received your letter of the 27th of March. Thirty persons on the staffs of the Schools attached to the chief London Hospitals, according to the last Home Office Report, held certificates allowing them to cut up animals alive without any anæsthetics whatever. They are distributed as follows:—

| | |
|---|---|
| University ... ... ... ... ... | 4 |
| King's ... ... ... ... ... | 3 |
| St. Bartholomew's ... ... ... ... | 10 |
| Guy's ... ... ... ... ... ... | 6 |
| St. Thomas's ... ... ... ... ... | 5 |
| St. Mary's ... ... ... ... ... | 2 |

If, nevertheless, you suggest that no cruelties take place, I must assume that you mean that nothing more than trifling inoculations are performed under these thirty persons' hands.

Now the pages of the *Journal of Physiology* are full of accounts of the most shocking vivisections, hardly any of which are performed under a better anæsthetic than choral or morphia, and many under curare alone, which is not an anæsthetic at all;—vivisections lasting for hours, during which the cut nerves of the animals are "stimulated" with electricity. Here, therefore, we have the certain fact that inconceivable suffering is inflicted by English vivisectors on "hapless dumb animals" together with the equally certain fact that thirty persons in London take out special certificates enabling them legally to inflict that suffering. Are we to suppose that the fortuitous contiguity of a hospital renders all these thirty vivisectors different from their fellow vivisectors whose laboratories have some building other than a hospital next door? What evidence have you that these particular vivisectors having obtained these certificates only use them for inoculations?—none other than the evidence of the very persons themselves whose conduct is impugned!

Perhaps you will say that the inspector reports these thirty to be guiltless of cruelties. But that amiable man makes a similar report about them *all*, whether connected with hospitals or not, and therefore, with the *Journal of Physiology* before us, his report becomes waste paper; and indeed he can hardly be expected to supply anything we can regard as "evidence" of what happens at times when, and places where, he is not present.

If seventy-seven people are seen going into a restaurant at supper time, if they stay there long enough for a hearty meal, and are observed to approach the cashier and pay the price of the repast, should thirty of them subsequently assert that they each took no more than a glass of water, the burden of proof would lie with THEM.—I am, Sir, yours faithfully,

Henry C. Burdett, Esq. STEPHEN COLERIDGE.

Mr. H. C. Burdett to Hon. Stephen Coleridge.

The Lodge, Porchester Square, W.
29th March, 1897.

Sir,—I have to point out that your letter of the 29th instant is no reply to mine of the 27th instant. It is certainly incumbent upon you to either supply the names of the laboratories which you stated in the *Times* to be attached to hospitals where "hapless dumb animals daily and hourly are slowly cut up alive without even the pretence of anæsthetics," or frankly to withdraw that statement.—I am, Sir, yours faithfully, Henry C. Burdett.

Hon. S. Coleridge,
7, Egerton Mansions, South Kensington.

The Hon. Stephen Coleridge to Mr. H. C. Burdett.

7, Egerton Mansions, S.W.
29th March, 1897.

Sir,—I have the honour to point out that my letter of the 29th was a courteous and patient answer in the most conclusive nature to your letter of the 27th. I have also to point out that I have taken some trouble to furnish you with facts relating to the financial connection between hospitals and laboratories which your thirty years of study had apparently overlooked. What properly is due, therefore, is not any withdrawal on my part, but some expressions of obligation on yours.—I am, Sir, your obedient servant,

Stephen Coleridge.

Mr. H. C. Burdett to Hon. Stephen Coleridge.

The Lodge, Porchester Square, W.
3rd April, 1897.

Sir,—In your letter of the 26th ultimo you state that the London Hospital and Guy's have Medical Schools attached to them, where vivisection is practised, that have received grants from Hospital Funds. The Treasurer of Guy's Hospital informs me "it is quite possible (though he does not know it as a fact) that prior to 1880 the Governors of Guy's Hospital did expend some of their surplus income arising from their estates on assisting the Medical and Surgical School with buildings." He can answer, however, that since 1882 "not one shilling of hospital money has been spent on School Buildings. The College and the new buildings have been built entirely out of moneys raised by the staff on the security of school fees." So much for Guy's Hospital.

As to the London Hospital, I refer you to Parliamentary Paper 330, being the last published return of "Experiments on Living Animals," which shows the number performed during the year 1895 under licences, from which you will find that no such experiments were performed at this hospital.

I have also to acknowledge the receipt of your second letter of March 29th. I cannot regard it as an "answer of a most conclusive nature" to my inquiries. You are said to have stated in a letter in the *Times* that in some laboratories attached to hospitals "hapless dumb animals daily and hourly are slowly cut up alive without even the pretence of anæsthetics." You ask me to infer the accuracy of your statements. The charges of Mr. Bryan and yourself are of far too serious a character to be allowed to rest upon inferences, and I ask again for definite information as to:—

1. The laboratories attached to hospitals in which animals are slowly cut up alive.
2. The laboratories attached to hospitals in which animals are cut up alive without the use of anæsthetics.
3. The hospitals to which such laboratories are attached.
4 The names of all the hospitals where the patients or any of them are treated as subjects for experimentation apart from the treatment of their maladies.

I am leaving England under doctors' orders for some weeks, but any reply addressed to Mr. P. A. Nairne, 3, Crosby Square, E.C., will receive prompt attention.—I am, Sir, yours faithfully, Henry C. Burdett.

Hon. Stephen Coleridge.

The Hon. Stephen Coleridge to Mr. H. C. Burdett.

7, Egerton Mansions, South Kensington, S.W.,
5th April, 1897.

Sir,—I have received your letter of the 3rd of April, 1897. The Treasurer of Guy's Hospital naturally confirms the information I gave you, including the date of my reference.

With regard to the London Hospital, the Parliamentary report No. 330 to which you refer me, states in its second paragraph that "the names of all those 'licensed places' to which licensees were accredited, are given in the tables." The London Hospital Medical School *was* such a "licensed place" during the period reviewed in this report. The report, therefore, in omitting as it does, this "licensed place" from its tables contains a gross error, and we may at once dismiss as utterly untrustworthy a Parliamentary paper drawn up with such culpable carelessness, and the information I gave you, both as regards Guy's and the London Hospital, remains unshaken.

With regard to my assertion as to animals being cut up alive without anæsthetics, my position is simple and impregnable. Their own publications, to which I have referred you, prove that the vivisectors in this country do slowly cut up animals alive without anæsthetics, and as long as they apply for and receive certificates for the purpose of cutting up animals alive without anæsthetics in the laboratories attached to hospitals, my Society will continue to assert that what they obtain licences to do, they do do.

You complain that such assertion is only based upon inference.

If a man takes out a licence year after year to drive a hansom cab, do you call it only an inference to assert upon that evidence that he does drive a hansom cab? We say with confidence that such evidence justifies anyone in calling that man a cab driver, and if a man takes out a licence to cut up animals alive without anæsthetics it equally justifies us in calling him an animal torturer. The burden of proof lies upon the first man to prove that he does not drive a hansom cab, and the burden of proof lies on the second man to prove that he does not torture animals.

I have already supplied you with the names of the laboratories attached to hospitals in which persons are licensed to cut up animals alive as slowly as they like, and as often as they like, without any anæsthetics whatever, and inasmuch as hospitals are dependent for their support chiefly upon humane persons, it is not surprising that, as the practice of vivisection has increased in these laboratories, the subscriptions have fallen away.—Your obedient servant,

H. C. Burdett, Esq. Stephen Coleridge.

I sincerely trust your sojourn abroad will entirely restore you to health.

[No further letter from Mr. Burdett himself was received. —Ed. *Z.*]

This, however, did not really complete the correspondence, which was continued as follows:

Mr. Percival A. Nairne to Hon. Stephen Coleridge.

3, Crosby Square, Bishopsgate, London, E.C.,
April 15th, 1897.

Sir,—Mr. Burdett being away from England, your letter to him date April 5th has, according to his directions, been forwarded to me, as before leaving he asked me to acknowledge any reply that might be received from you to his letter of April 3rd.

I have read through the correspondence.

The information received by Mr. Burdett from the treasurer of Guy's Hospital is, in my view, not a confirmation of the original charge that "the practice of vivisection is carried on in medical schools attached to hospitals and receiving grants from the Hospital funds." The treasurer's information was that the medical school attached to Guy's Hospital has received no grants from the Hospital funds since 1882. The charge refers to medical schools now receiving grants from Hospital funds.

I cannot myself condemn the Parliamentary Report No. 330 as "utterly untrustworthy and drawn up with culpable carelessness." I observe that you refer to it as an authority in your letter of March 29th. The London Hospital Medical College is mentioned in foot-notes on pages 13 and 15 as a place where experiments might be performed, though it does not appear that any experiments were performed there.

Mr. Burdett in his letter to you of April 3rd asked for definite information on four points.

In reply, you again ask him to draw inferences which he has already, and I think rightly, declined to do, having regard to the serious character of the charges made. Your illustrations are forcible, but analogy is not usually accepted as sufficient proof of charges of this nature.—I am, Sir, your obedient servant, Perceval A. Nairne.

The Hon. Stephen Coleridge to Mr. Percival A. Nairne.

The Ford, Greywell, Hampshire,
April 17th, 1897.

Sir,—My analogies have been introduced not as proofs of facts, but as aids for the formation in Mr. Burdett's mind of a due estimate of the weight of my inferences.

Persons are often hanged upon inferences, for murderers do not generally wait till they can procure an eye-witness of their crime.

In human affairs inference is commonly the basis of right judgment, as in this case.—Your obedient servant,

Stephen Coleridge.

We hope that our readers will be able to extract from these somewhat wordy letters the true meaning of this correspondence. On receiving a letter stating that vivisection is now carried on in medical schools attached to hospitals and receiving grants from hospital funds, Mr. Burdett immediately wrote to ask the names of such medical schools, and also asking the names of the laboratories to which Mr. Coleridge referred when he said that in laboratories attached to hospitals animals were daily and hourly cut up alive without even the pretence of anæsthetics.

To the first question Mr. Coleridge answered by giving the names of the London Hospital and Guy's. On making inquiries into the matter, Mr. Burdett found that so far from this being correct Guy's Hospital Medical School had received no assistance from hospital funds since the year 1882, so that the suggestion that money subscribed to Guy's Hospital would be used to support a medical school in which vivisection was practised was distinctly misleading. In regard to the other assertion that "hapless dumb animals daily and hourly are slowly cut up alive without even the pretence of anæsthetics" in laboratories attached to hospitals, Mr. Coleridge gives a list of the hospitals in London attached to which are persons who hold certificates allowing them, as he says, "to cut up animals alive without any anæsthetics."

When, however, he is pressed to state at which of these laboratories these things are done he falls back on inference and analogy, and fails to point out a single hospital medical school, either assisted by hospital funds or not, where such proceedings take place. Doubtless all our readers will admire the ingenuity of Mr. Coleridge's illustrations and inferences, almost as much as they will wonder at the levity with which they are introduced into so serious a discussion. The point, however, is that they are but inferences after all. If we come to fact we find quite another explanation of these licences to operate without anæsthetics.

On April 29th, in the House of Commons, Mr. Weir asked the Secretary of State for the Home Department if he would state the number of persons licensed to practice vivisection who held certificates to dispense with anæsthetics. Sir Matthew White Ridley stated in reply that the number in England was 86, but added that "in giving the hon. member these figures he might remind him that the certificate in question was never given for operations involving serious pain, but only for such operations as inoculations or hypodermic injections."

This is a responsible statement made by a responsible Minister of the Crown from his place in Parliament, and we confess to preferring it to the irresponsible inferences and analogies made by irresponsible persons who take up the subject as a fad.

In view then of this statement in Parliament, all the talk about cutting animals up alive without even a pretence of anæsthetics must be looked upon as sheer nonsense; and we can but express our surprise that persons who consider themselves kind-hearted should make such wild assertions at a moment when it must be obvious that their only effect must be to deprive the hospitals of London of funds which they sorely need for the relief of suffering.—Ed. *T. H.*

